# Circulating YKL-40 Level, but not *CHI3L1* Gene Variants, Is Associated with Atherosclerosis-Related Quantitative Traits and the Risk of Peripheral Artery Disease

**DOI:** 10.3390/ijms151222421

**Published:** 2014-12-04

**Authors:** Semon Wu, Lung-An Hsu, Shih-Tsung Cheng, Ming-Sheng Teng, Ching-Hua Yeh, Yu-Chen Sun, Hsuan-Li Huang, Yu-Lin Ko

**Affiliations:** 1 Department of Research, Taipei Tzu Chi Hospital, Buddhist Tzu Chi Medical Foundation, Taipei 23142, Taiwan; E-Mails: semonwu@yahoo.com.tw (S.W.); vincent@tzuchi.com (M.-S.T.); aethiopica@gmail.com (C.-H.Y.); huang304@ms11.hinet.ne (H.-L.H.); 2 Department of Life Science, Chinese Culture University, Taipei 11114, Taiwan; 3 The First Cardiovascular Division, Department of Internal Medicine, Chang Gung Memorial Hospital and Chang Gung University College of Medicine, Taoyuan 33305, Taiwan; E-Mail: hsula@adm.cgmh.org.tw; 4 The Division of Cardiology, Department of Internal Medicine and Cardiovascular Center, Taipei Tzu Chi Hospital, Buddhist Tzu Chi Medical Foundation, Taipei 23142, Taiwan; E-Mail: chengshihtsung@gmail.com; 5 Department of Laboratory Medicine, Chang Gung Memorial Hospital, Taipei 33305, Taiwan; E-Mail: sun036@adm.cgmh.org.tw; 6 School of Medicine, Tzu Chi University, Hualien 97071, Taiwan

**Keywords:** circulating YKL-40 level, *CHI3L1* gene variants, peripheral artery disease, association study, haplotype, risk factor

## Abstract

YKL-40, a pleotropic cytokine, is emerging as a risk factor and a prognostic predictor of atherosclerotic cardiovascular disease. We attempted to elucidate the genetic, clinical and biochemical correlates of circulating YKL-40 level and, by combining it with *CHI3L1* gene variants, with the risk and long-term mortality of peripheral artery disease (PAD). Plasma YKL-40 concentrations were measured in 612 Taiwanese individuals who had no clinically overt systemic disease. Clinical parameters, *CHI3L1* gene promoter variants and 18 biomarker levels were analyzed. Eighty-six PAD patients were further enrolled for analysis. Significant associations were found between *CHI3L1* genotypes/haplotypes and YKL-40 levels for the health examination subjects (smallest *p =* 8.36 × 10^−7^ for rs4950928 and smallest *p* = 1.72 × 10^−10^ for haplotype TGG) and also for PAD patients. For the health examination subjects, circulating YKL-40 level, but not *CHI3L1* gene variants, were positively associated with age, smoking, and circulating levels of triglyceride, lipocalin 2 and multiple inflammatory biomarkers and negatively associated with low-density-lipoprotein cholesterol levels. Circulating YKL-40 level is also significantly associated with the risk of PAD (*p* = 3.3 × 10^−23^). Circulating YKL40 level, but not *CHI3L1* gene promoter variants, is associated with the risk of PAD in Taiwanese. The association of YKL-40 levels with multiple quantitative traits relating to the risk of PAD may provide a molecular basis linking YKL-40 to atherosclerotic cardiovascular disease.

## 1. Introduction

YKL-40, a 40 kDa chitin-binding glycoprotein without chitinase activity, is an acute phase protein expressed by a variety of cells, including macrophage. It has been shown to act as an important regulator of acute and chronic inflammation and tissue remodeling [1,2,3,4]. YKL-40 seems especially involved in activation of the innate immune system and is highly up-regulated in distinct subsets of macrophages in the atherosclerotic plaques [5]. The suppression of atherosclerosis in apolipoprotein E knockout mice by lentivirus-mediated *CHI3L1* gene silencing suggests a role of YKL-40 on plaque progression and as a candidate therapeutic target in atherosclerosis [6]. Substantial evidence indicates a pathogenic role of YKL-40 in endothelial dysfunction and the earliest part of the atherosclerotic process leading to disease progression and manifest cardiovascular disease [1,2,3]. However, the molecular processes inducing YKL-40 and the precise functions of YKL-40 have not yet been identified.

Circulating YKL-40 levels increase in patients with acute infection and chronic inflammation. Recent studies have reported that elevated levels of plasma YKL-40 are proportional with the homeostasis model assessment of insulin resistance (HOMA-IR) in type 2 diabetes subjects [7,8]. Several clinical studies documented elevated YKL-40 levels in patients with cardiovascular disease, including coronary artery disease [9,10] peripheral artery disease (PAD) [11] and stroke [12]. An association was noted between higher YKL-40 level and increased mortality in elderly persons and stable coronary artery disease [13,14,15,16]. The *CHI3L1* gene, encoding the YKL-40 protein, is located at chromosome 1q31–q32, consisting of 10 exons and spans about 8 kb of genomic DNA. Significant and constant associations between promoter region variants of the *CHI3L1* gene with YKL-40 levels have been reported in both the general population and disease states [9,10,11,12,17,18,19,20]. Previous studies have shown the association of the *CHI3L1* gene promoter region polymorphisms with stroke, schizophrenia, personality trait, atrial fibrillation, asthma and reduced lung function [18,19,20,21]. In contrast, controversial results were reported regarding the association of *CHI3L1* gene variants with atherosclerotic cardiovascular diseases [9,10,12]. We conducted this study in an attempt to elucidate the associations of YKL-40 levels and *CHI3L1* gene variants with various metabolic traits, adipokine levels and inflammatory marker levels and the risk and long-term mortality of PAD in Taiwanese.

## 2. Results

### 2.1. Clinical and Biochemical Characteristics

A summary of demographic features, clinical profiles, and levels of biomarkers for the studied health examination participants is provided in Table 1. No significant deviation from the Hardy–Weinberg equilibrium was detected for the studied polymorphisms (*p* = 0.992, 0.959 and 0.705 for SNPs rs10399931, rs10399805 and rs4950928, respectively) (Table S1). All three polymorphisms were found to have strong pairwise linkage disequilibrium (Table S2).

**Table 1:** Baseline characteristics of the health examination subjects.

Baseline Characteristics	Total	Men	Women	*p* Value
Number	612	323	289	
Age (years)	46.2 ± 10.0	45.6 ± 10.0	47.0 ± 10.0	0.082
Systolic BP (mm Hg)	113.1 ± 16.1	114.0 ± 14.3	112.1 ± 17.8	0.169
Diastolic BP (mm Hg)	75.0 ± 10.0	76.8 ± 9.7	73.1 ± 10.0	<0.001
Mean BP (mm Hg)	87.7 ± 11.2	89.2 ± 10.4	86.1±11.8	0.001
Total cholesterol (mg/dL)	198.8 ± 36.4	200.7 ± 36.9	196.6 ± 35.9	0.164
HDL-cholesterol (mg/dL)	55.3 ± 14.3	49.8 ± 11.9	61.3 ± 14.3	<0.001
LDL-cholesterol (mg/dL)	116.1 ± 32.9	118.4 ± 33.8	113.5 ± 31.6	0.064
Triglyceride (mg/dL)	142.4 ± 118.5	171.8 ± 147.2	109.9 ± 60.6	<0.001
Body mass index (kg/m^2^)	24.3 ± 3.5	24.9 ± 3.1	23.6 ± 3.7	<0.001
Diabetes mellitus (%)	5.1	5.9	4.2	0.330
Current smokers (%)	19.3	33.1	3.8	<0.001
Fasting plasma glucose (mg/dL)	96.2 ± 22.5	98.9 ± 25.7	93.2 ± 17.7	0.002
Fasting serum insulin (μU/mL)	9.2 ± 4.9	9.8 ± 5.5	8.6 ± 3.9	0.003
HOMA-IR index	2.2 ± 1.4	2.4 ± 1.6	2.0 ± 1.1	<0.001
QUICKI	0.35 ± 0.25	0.34 ± 0.25	0.35 ± 0.24	<0.001
Adiponectin (mg/L)	7.2 ± 5.2	5.4 ± 4.0	9.1 ± 5.6	<0.001
Resistin (ng/mL)	18.6 ± 14.3	17.9 ± 12.2	19.2 ± 16.4	0.234
Lipocalin 2 (ng/mL)	80.4 ± 52.0	83.9 ± 59.8	76.5 ± 41.7	0.078
CRP (mg/L)	1.08 ± 1.39	1.10 ± 1.38	1.07 ± 1.40	0.186
Fibrinogen (mg/dL)	264.9 ± 70.3	262.8 ± 72.4	267.4 ± 68.0	0.424
sE-selectin (ng/mL)	53.3 ± 25.1	60.0 ± 25.8	45.7 ± 21.8	<0.001
sP-selectin (ng/mL)	139.9 ± 117.0	154.5 ± 131.9	123.5 ± 95.5	0.001
SAA (mg/L)	6.1 ± 15.4	7.0 ± 19.6	5.1 ± 8.9	0.131
sICAM1 (ng/mL)	241.3 ± 110.6	243.9 ± 109.2	238.4 ± 112.3	0.583
sVCAM1 (ng/mL)	491.0 ± 131.9	494.2 ± 148.7	487.4 ± 110.3	0.652
MMP1 (pg/mL)	467.2 ± 1151.8	338.9 ± 545.4	609.7 ± 1560.8	0.673
MMP2 (ng/mL)	126.5 ± 40.9	123.2 ± 41.2	130.1 ± 40.4	0.038
MMP-9 (ng/mL)	143.5 ± 111.9	155.1 ± 115.8	130.6 ± 105.9	0.007
MCP1 (pg/mL)	73.0 ± 58.3	78.0 ± 66.1	67.4 ± 47.6	0.010
sTNFRII (pg/mL)	3270.9 ± 947.5	3328.1 ± 981.5	3207.4 ± 905.7	0.102
Creatinine (mg/dL)	0.99 ± 0.46	1.12 ± 0.44	0.84 ± 0.44	<0.001
eGFR (mL/min/1.73 m^2^)	81.03 ± 14.94	79.38 ± 13.55	82.93 ± 16.21	0.007
YKL-40 (ng/mL)	92.37 ± 90.32	95.08 ± 101.50	89.14 ± 74.94	0.283
8-OHdG/creatinine (ng/mg)	38.12 ± 24.44	35.65 ± 24.97	40.9 ± 23.56	0.008

BP, blood pressure; HDL, high-density lipoprotein; LDL, low-density lipoprotein; HOMA-IR, homeostasis model assessment of insulin resistance; QUICKI, quantitative insulin sensitivity check index; CRP, C-reactive protein; SAA, serum amyloid A; sE-selectin, soluble E-selectin; sP-selectin, soluble P-selectin; sICAM1, soluble intercellular adhesive molecule 1; sVCAM1, soluble vascular cell adhesive molecule 1; MMP1, matrix metalloproteinase 1; MMP2, matrix metalloproteinase 2; MMP9, matrix metalloproteinase 9; MCP-1, Monocyte chemotactic protein-1; sTNFRII, soluble tumor necrosis factor-alpha receptor 2; eGFR, estimated glomerular filtration rate; 8-OHdG: 8-hydroxy-2-deoxyguanosine. Continuous variables are presented as mean ± SD. HDL-C, LDL-C, Total cholesterol, Triglyceride, CRP, SAA, sICAM1, sVCAM1, sE-selectin, sP-selectin, MMP1, MMP2, MMP9, YKL-40, MCP1, and sTNFRII values were logarithmically transformed before statistical testing to meet the assumption of normal distributions; however, the untransformed data are shown. BP levels and lipid variables were analyzed with the exclusion of subjects using antihypertensive drugs and/or lipid-lowering agents. Fasting plasma glucose and insulin and HOMA-IR index were analyzed with the exclusion of anti-diabetic medications. CRP level was calculated with the exclusion of subjects with CRP levels above10 mg/L.

**Table 2:** Demographics of PAD patients with or without mortality.

Demographics		Total (*n* = 86)	Survival ( *n* = 76)	Mortality (*n* = 10)	*p* Value
Age		71.65 ± 10.90	71.89 ± 10.05	71.67 ± 16.00	0.951
Sex (male/female)		50/36	44/32	6/4	1.000
Body mass index		23.94 ± 3.82	24.11 ± 3.69	23.46 ± 4.50	0.321
Smoking		33.3%	33.8%	30.0%	1.000
Diabetes mellitus		73.3%	75.0%	60.0%	0.447
Dyslipidemia		42.2%	43.8%	30.0%	0.507
Hypertension		84.9%	86.8%	70.0%	0.172
Congestive heart failure		17.4%	17.1%	20.0%	1.000
Stroke		15.1%	14.5%	20.0%	0.644
End stage renal disease		41.2%	38.7%	60.0%	0.305
Coronary artery disease		55.8%	56.6%	50.0%	0.744
Rutheford grade	3: severe claudication	22.6%	24.3%	10%	0.443
	≥4: critical limb ischemia	77.4%	75.7%	90%	
YKL-40 (ng/mL)		406.74 ± 286.66	405.19 ± 299.00	418.47 ± 175.90	0.408
rs4950928 G-allele carriers		29.1%	30.3%	20%	0.502

### 2.2. Associations between YKL-40 Levels and Clinical and Biochemical Correlates

The associations between YKL-40 levels and clinical and biochemical correlates are shown in Table 3 and Table 4. Circulating YKL-40 level was positively associated with age, circulating levels of triglyceride, lipocalin 2, and multiple inflammatory biomarkers including C-reactive protein (CRP), sE-selectin, sVCAM1 and sTNFRII and negatively associated with LDL-cholesterol levels (Table 3). Further, we analyzed the association between YKL-40 levels and the presence or absence of several risk factors for cardiovascular disease. There were higher plasma YKL-40 levels among current smokers than non-current smokers after the adjustment of age and sex (Table 4). Higher plasma YKL-40 level was associated in hypertension subjects, but it disappeared after the adjustment of age and sex. In contrast, there was no evidence of an association of YKL-40 levels with diabetic status and body mass parameters.

**Table 3:** Association between YKL40 levels and measurable risk factors in health examination subjects.

Clinical Biochemical Parameters		Unadjusted		Adjusted for Age and Sex		
		*r*	*p* Value	*r*	*p* Value	Adjusted *p*
Anthropology	Age	0.346	4.85 × 10^−18^	–	–	–
	Body mass index	0.022	0.596	−0.008	0.853	NS
	Waist circumference	0.088	0.032	0.029	0.481	NS
Blood pressure	Systolic BP	0.122	0.005	−0.015	0.734	NS
	Diastolic BP	0.067	0.125	−0.004	0.934	NS
Glucose metabolism	Fasting plasma glucose	0.082	0.049	0.069	0.099	NS
	Fasting serum insulin	0.064	0.127	0.074	0.077	NS
	HOMA-IR index	0.085	0.041	0.094	0.024	NS
	QUICKI	−0.062	0.136	−0.067	0.109	NS
Lipid profiles	Total cholesterol	0.008	0.843	−0.050	0.234	NS
	LDL-cholesterol	−0.083	0.046	−0.146	4.34 × 10^−4^	0.013
	HDL-cholesterol	−0.062	0.136	−0.065	0.121	NS
	Triglyceride	0.226	3.95 × 10^−8^	0.220	9.4 × 10^−8^	2.82 × 10^−6^
Renal function	Microalbumin/creatinine	0.123	0.003	0.096	0.021	NS
	eGFR	−0.186	3.25 × 10^−5^	1.9 × 10^−4^	0.997	NS
Inflammation marker	CRP	0.172	3.25 × 10^−5^	0.142	0.001	0.003
	Fibrinogen	0.127	0.002	0.058	0.158	NS
	sE-selectin	0.147	3.6 × 10^−4^	0.144	0.001	0.003
	sP-selectin	−0.023	0.584	−0.047	0.259	NS
	sVCAM1	0.211	2.69 × 10^−7^	0.155	1.79 × 10^−4^	0.005
	sICAM1	0.107	0.010	0.094	0.023	NS
	TNFRII	0.196	1.81 × 10^−6^	0.150	2.82 × 10^−4^	0.008
	MCP1	0.037	0.372	0.022	0.600	NS
	MMP1	−0.032	0.443	−0.023	0.572	NS
	MMP2	0.052	0.211	0.008	0.854	NS
	MMP9	0.014	0.735	0.038	0.366	NS
	SAA	−0.005	0.910	0.007	0.870	NS
Adipokines	Leptin	6.57 × 10^−5^	0.999	0.023	0.571	NS
	Resistin	0.116	0.006	0.115	0.006	NS
	Lipocalin2	0.149	3.82 × 10^−4^	0.162	1.09 × 10^−4^	0.003
	Adiponectin	0.069	0.092	0.056	0.173	NS
Oxidative stress	Homocysteine	0.108	0.008	0.100	0.016	NS
	8-OHdG/creatinine	0.077	0.062	0.046	0.262	NS

Abbreviations as Table 1. NS: No Significance; 8-OHdG: 8-hydroxy-2-deoxyguanosine. BP levels and lipid variables were analyzed with the exclusion of subjects using antihypertensive drugs and/or lipid-lowering agents. Fasting plasma glucose and insulin and HOMA-IR index were analyzed with the exclusion of anti-diabetic medications. CRP level was calculated with the exclusion of subjects with CRP levels above 10 mg/L. Microalbuminuria/creatinine was calculated with the exclusion of subjects with Microalbuminuria/creatinine > 300. Adjusted *p* values were computed by utilizing the Bonferroni method.

**Table 4:** Association between YKL40 levels and atherosclerotic risk factors in health examination subjects.

Atherosclerotic Risk Factors		YKL40 Levels (Means ± SD) (N)	*p* Value ^a^	*p* Value ^b^
Current smoker	Noncurrent	88.64 ± 74.09 (472)	0.118	0.010
	Current	107.42 ± 137.05 (177)		
Hypertension	Without	88.02 ± 90.44 (472)	0.001	0.499
	With	109.90 ± 88.09 (117)		
Diabetes mellitus	Nil	90.59 ± 87.87 (558)	0.102	0.557
	Yes	124.37 ± 124.16 (31)		
Obesity	Nonobese	93.29 ± 103.38 (353)	0.281	0.878
	Obese	91.00 ± 66.33 (236)		
Metabolic syndrome	Nil	90.89 ± 93.09 (478)	0.099	0.674
	Yes	98.74 ± 77.32 (111)		
Insulin resistance	Nil	87.82 ± 75.68 (441)	0.063	0.127
	Yes	105.92 ± 123.46 (148)		

^a^: Not adjusted; ^b^: Multiple linear regression adjusted for age and sex.

### 2.3. Associations of the CHI3L1 Genotypes/Haplotypes with Circulation Levels of YKL40 in Subjects from Health Examination and in PAD Patients

To determine whether the *CHI3L1* genotypes affected circulating YKL-40 levels, three SNPs were analyzed. Our results showed that genetic variants in the promoter region of the *CHI3L1* gene were significantly associated with YKL-40 levels in Taiwanese (Table 5). After adjusting for clinical covariates, significant associations with YKL-40 level were observed for two polymorphisms, rs10399931 and rs4950928, using an additive inheritance model. The differences remained significant even after the use of stringent Bonferroni correction for multiple tests in subjects from health examination (*p* = 1.87 × 10^−5^ and *p* = 5.02 × 10^−6^, for rs10399931 and rs4950928, respectively) and PAD patients (*p* = 1.16 × 10^−3^ for rs4950928). With a dominant model, minor alleles of rs10399931 and rs4950928 were found to be associated with a lower YKL-40 level. The differences also remained significant even after the use of stringent Bonferroni correction for multiple tests (*p* = 3.6 × 10^−9^ and *p* = 1.37 × 10^−9^, respectively for subjects from health examination and *p* = 0.006 and *p* = 3.97 × 10^−5^, respectively, for PAD patients). Because single SNP regression demonstrated that multiple sites within or near the *CHI3L1* gene significantly affected YKL-40 level, haplotypes were inferred to capture possible allelic associations. In the present investigation, 4 common haplotypes (≥2% frequency) were derived from three SNPs, accounting for 98.19% of all inferred haplotypes. In haplotype analysis, two haplotypes inferred from three SNPs (CGC and TGG) were found to be associated with YKL-40 level (Table 6). The differences also remained significant even after the use of stringent Bonferroni correction for multiple tests in subjects from health examination (*p* = 7.12 × 10^−8^ and *p* = 6.88 × 10^−8^, for CGC and TGG, respectively) and PAD patients (*p* = 4.24 × 10^−4^ for TGG).

**Table 5:** Association between *CHI3L1* genotypes and YKL-40 levels

CHI3L1 Genotypes		Health Examination Subjects			PAD		
		YKL-40 Level Means ± SD (N)	*p* Value	Adjusted *p*	YKL-40 Level Means ± SD (N)	* p Value	Adjusted *p*
rs10399931	CC	104.77 ± 110.27 (232)	3.12 × 10^−6^	1.87 × 10^−5^	548.74 ± 356.70 (30)	0.011	0.066
	CT	82.79 ± 65.46 (267)			346.76 ± 197.28 (40)		
	TT	74.98 ± 88.40 (79)			290.43 ± 232.65 (16)		
	CC	104.77 ± 110.27 (232)	6 × 10^−10^	3.6 × 10^−9^	548.74 ± 356.70 (30)	0.001	0.006
	CT + TT	81.01 ± 71.29 (346)			330.67 ± 207.41 (56)		
rs10399805	AA	102.67 ± 114.85 (42)	0.328	1.00	568.36 ± 417.22 (10)	0.072	0.432
	AG	87.79 ± 71.23 (230)			452.92 ± 282.13 (32)		
	GG	93.14 ± 98.01 (308)			336.42 ± 236.43 (44)		
	AA + AG	90.09 ± 79.44 (272)	0.362	1.00	480.40 ± 317.60 (42)	0.074	0.444
	GG	93.14 ± 98.01 (308)			336.42 ± 236.43 (44)		
rs4950928	CC	100.99 ± 99.59 (404)	8.36 × 10^−7^	5.02 × 10^−6^	467.63 ± 302.62 (61)	1.94 × 10^−4^	1.16 × 10^−3^
	CG	71.64 ± 56.42 (154)			283.39 ± 174.75 (21)		
	GG	59.65 ± 53.66 (21)			125.73 ± 77.11 (4)		
	CC	100.99 ± 99.59 (404)	2.29 × 10^−10^	1.37 × 10^−9^	467.63 ± 302.62 (61)	6.61 × 10^−6^	3.97 × 10^−5^
	CG + GG	70.20 ± 56.09 (175)			258.17 ± 172.25 (25)		

*p* Value, adjusted for age, sex, BMI, current smoker, use of antihypertensive, antidiabetic and lipid lowering drugs; * p value, adjusted for age, sex, BMI, current smoker, antihypertensive and lipid lowering drugs; Adjusted *p* values were computed by utilizing the Bonferroni method.

### 2.4. Associations Analysis of YKL-40 with Clinical Parameters and Various Biomarker Levels

The associations between *CHI3L1* genotypes and clinical and biochemical correlates are shown in Table S3. Although subjects with the GG genotype of rs4950928 have significantly lower waist circumference and eGFR and higher microalbumin/creatinine, sVCAM1, MMP2 and leptin levels in initial analysis, none of them remain significant after Bonferroni correction for multiple tests (Table S3).

### 2.5. Comparison of Circulating YKL-40 Level and CHI3L1 Gene Variants between Subjects from Health Examination and PAD Patients

**Figure 1:**
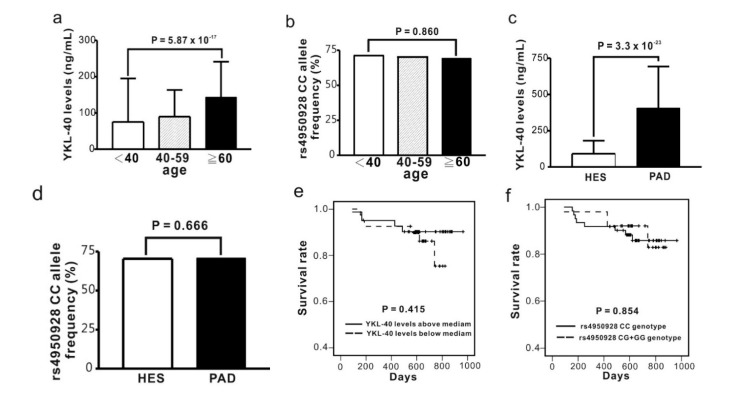
Association of circulating YKL-40 level and *CHI3L1* gene variants with age, disease status and long-term outcome of peripheral artery disease (PAD). For health examination subjects (HES), YKL-40 levels (**a**) but not *CHI3L2* gene variants (**b**) are significantly associated with age; PAD patients have higher YKL-40 levels compared to HES (**c**); while no significant difference was noted between the two study groups in the *CHI3L1* genotype frequencies (**d**); Kaplan-Meier survival curve showed no difference for long-term mortality in PAD patients between subgroups of YKL-40 levels above or below the median (**e**) and between *CHI3L1* genotypes (**f**).

In different age subgroups of health examination subjects, we found that YKL-40 level was positively associated with age (*p* = 5.87 × 10^−17^) (Figure 1a). Significantly higher YKL-40 levels, but not different *CHI3L1* genotype frequencies, were found in PAD patients in comparison with subjects from health examination (*p* = 3.3 × 10^−23^) (Figure 1c). In contrast, no significant difference in the genotype frequencies of the *CHI3L1* gene variants was noted in different age subgroups of both populations (Figure 1b,d). In addition, no significant difference for YKL-40 levels and *CHI3L1* genotypes was found on long-term mortality of PAD patients (Table 2, Figure 1e,f).

**Table 6:** Association of *CHI3L1* locus haplotypes with YKL-40 level.

Haplotypes	Health Examination Subjects							PAD			
	SNP1	SNP2	SNP3	Frequency	Estimate	*p* Value	Adjusted *p*	Frequency	Estimate	* p Value	Adjusted *p*
H1	C	G	C	35.22%	0.172	1.78 × 10^−8^	7.12 × 10^−8^	27.91%	0.248	0.037	0.148
H2	C	A	C	26.93%	0.049	0.136	0.544	30.23%	0.179	0.086	0.344
H3	T	G	C	21.04%	−0.067	0.066	0.264	25.00%	0.037	0.343	1.000
H4	T	G	G	15.30%	−0.252	1.72 × 10^−10^	6.88 × 10^−8^	16.86%	−0.560	1.06 × 10^−6^	4.24 × 10^−6^

SNP1: rs10399931, SNP2: rs10399805, SNP3: rs4950928. Coefficients and *p* values were estimated based on haplotype trend regression analysis implemented in the HelixTree program. The selected haplotype compared to all unselected haplotypes; *p* value, adjusted for age, sex, BMI, current smoker, use of antihypertensive, antidiabetic and lipid lowering drugs; * p Value, adjusted for age, sex, BMI, current smoker, antihypertensive and lipid lowering drugs; Adjusted *p* values were computed by utilizing the Bonferroni method.

## 3. Discussion

This investigation evaluated various genetic and non-genetic correlates of YKL-40 levels in Taiwanese. The most important point to highlight after conducting this study is that although *CHI3L1* gene promoter variants showed highly significant association with YKL-40 levels, only the latter revealed a significant association with multiple atherosclerosis-related traits and the risk of PAD. In addition to previously reported associations with age and triglyceride and CRP levels, we first reported that circulating levels of sE-seletin, sVCAM1, sTNFRII and lipocalin 2 were significantly associated with YKL-40 levels even after the use of stringent Bonferroni correction for multiple tests. These results provide further evidence that YKL-40 levels are associated with the risk of atherosclerotic cardiovascular diseases.

### 3.1. Association between YKL-40 Levels and CHI3L1 Gene Promoter Variants and the Risk of Atherosclerotic Cardiovascular Disease

Both circulating levels of YKL-40 and its coding gene *CHI3L1* promoter variants have been shown to be associated with multiple phenotypes and disease states, suggesting their important role in the molecular mechanism of various pathological conditions. However, their importance in atherosclerotic cardiovascular disease seemed to be much more diverse. Both YKL-40 levels and *CHI3L1* gene variants have been associated with the risk of stroke, while only the former was found associated with coronary artery disease. No evidence of an association between *CHI3L1* gene variants and coronary artery disease has been reported [9,10]. Batinic *et al.* [11] showed higher YKL-40 levels in PAD patients, however, the role of *CHI3L1* gene variants on PAD has not been investigated before. The consistent association of YKL-40 levels with atherosclerotic cardiovascular disease suggests its importance in a pathogenic role in atherosclerosis. In this investigation, in contrast to YKL-40 levels, we found no evidence of an association between the studied promoter variants and atherosclerosis-related quantitative traits and the risk of PAD, especially after Bonferroni correction for multiple tests. These results suggested the influence of baseline YKL-40 levels due only to *CHI3L1* promoter polymorphisms may not be large enough to alter the risk of most atherosclerotic cardiovascular diseases. We further found no evidence of an association of YKL-40 level and *CHI3L1* promoter variants with long term mortality of PAD, however, the possibility of the small sample size of PAD patients resulting in the absence of this association cannot be excluded.

### 3.2. Association of Clinical Parameters with YKL-40 Level

Previous studies have shown significant association of YKL-40 levels with conventional cardiovascular risk factors, including age, hypertension, diabetes, dyslipidemia and insulin resistance. Similar to previous reports [13,15,16,22], our data found age, but not gender, is significantly associated with YKL-40 levels that outrank all modifiable cardiovascular risk factors. Increasing age was also known as a potential risk factor of Alzheimer’s disease. Rosen *et al.* [23] found that Alzheimer’s disease patients had increased CSF levels of YKL-40 of approximately twice that of controls who had normal CSF profiles of core Alzheimer’s disease biomarkers. Antonell *et al.* [24] also detected that YKL0-40 level had significant correlation with t-tau and p-tau level in the predementia Alzheimer’s disease continuum and preclinical Alzheimer’s disease. In addition, higher YKL-40 levels were noted in current smokers in our study. We also confirmed the significant association between elevated YKL-40 and triglyceride levels, which was consistently reported before [7,11,12,16,22]. Thomsen *et al.* revealed higher total cholesterol and LDL-cholesterol levels with increased YKL-40 levels, which is different from our results showing negative association of LDL-cholesterol levels with YKL-40 levels [22]. Increased insulin resistance and microalbuminuria with increasing YKL-40 levels have also been described [12,22,25]. Increased blood pressure and incidence of hypertension was noted in other reports [15,16,22]. In this investigation, we found only a trend of higher YKL-40 levels in subjects with hypertension, diabetes mellitus and insulin resistance with the association of hypertension being predominantly age-related. Significantly higher HOMA-IR index and microalbuminuria with higher YKL-40 levels were also noted which subsided after Bonforreni correction for multiple tests. In brief, with the exception of association with lower LDL cholesterol level, higher YKL-40 levels in our study were associated with more atherosclerosis-related quantitative traits and a higher risk for atherosclerotic cardiovascular disease.

### 3.3. Roles of Association between YKL-40 Level and Biomarker Levels

In this investigation, we further analyzed the association of YKL-40 with various inflammatory and oxidative stress biomarkers and adipokines. YKL-40 is derived from acute and chronic inflammation associated with macrophages and adipocytes. YKL-40 seems to be an emerging biomarker in cardiovascular disease associated with inflammation and is a novel adhesion and migrating factor for vascular cell and plays a role in endothelial dysfunction [2,26]. Several reports have found higher circulating level of YKL-40 associated with high CRP, BNP, IL6, MCP1 levels and higher number of CD4/CD8 ratio in the circulation [13,14,16,22,27,28,29,30,31]. In this investigation, we found elevated YKL-40 levels have moderate to weakly positive association with higher levels of a lot of inflammatory markers, including CRP, sTNFRII, sE-selectin, and sVCAM1, supporting previous suggestion of the importance of YKL-40 in the roles of acute and chronic inflammation related cardiovascular diseases. Stromal vascular fraction cells of adipose tissue are considered a source of inflammation-related molecules. Expression levels of YKL-40, one of the non-traditional adipokines, in stromal vascular fraction cells from visceral adipose tissue were higher than in those from subcutaneous adipose tissue [32]. Lipocalin 2 is an inflammatory marker, an adipokine and also a marker of acute renal injury [33]. Our data showed a significant association of circulating YKL-40 and lipocalin 2 levels. These results revealed that YKL-40 is highly correlated with acute and chronic inflammation and renal dysfunctions that were also risk factors for future cardiovascular events.

## 4. Material and Methods

### 4.1. Study Population

A total of 612 Han Chinese subjects (323 men with a mean age of 45.6 ± 10.0 years and 289 women with a mean age of 47.0 ± 10.0 years) were recruited during routine health examinations between October 2003 and September 2005 at the Chang Gung Memorial Hospital. The studied subjects, responded to a questionnaire on their medical history and lifestyle characteristics, underwent a physical examination that involved measurement of height, weight, waist and hip circumference, and blood pressure in the sitting position after 15 min of rest. Fasting blood samples were obtained from each subject. Exclusion criteria included age below 18 years old, a history of myocardial infarction, stroke or transient ischemic attack, cancer, and current renal or liver disease. The clinical characteristics and biometrics of the study population are summarized in Table 1. Hypertension, obesity, current smokers and metabolic syndrome were defined as previously reported [34]. All the participants provided written informed consent and the studies were approved by the Ethics Committee of the Chang Gung Memorial Hospital (92-315, 6 May 2003).

Eighty-six consecutive hospitalized PAD patients without a known history of malignancy and receiving percutaneous transluminal angioplasty for advanced symptomatic PAD or critical limb ischemia of the lower extremities were enrolled for analysis (Table 2). All the participants provided written informed consent and the studies were approved by the Ethics Committee of the Taipei Tzu-Chi Hospital (02-M04-038, 8 December 2013).

### 4.2. Genomic DNA Extraction and Genotyping

Genomic DNA was extracted as reported previously [35]. Three single nucleotide polymorphisms (SNPs) at the promoter region of the *CHI3L1* gene were chosen in this study (Table S1). Genotyping for the SNPs rs10399931, rs10399805, and rs4950928 were performed using TaqMan SNP Genotyping Assays obtained from Applied Biosystems (ABI, Foster City, CA, USA).

### 4.3. Laboratory Examination

Before starting the study, all participants underwent an initial screening assessment that included medical history, vital signs and measurement of lipid variables and novel risk factors. A total of 15 mL of venous blood was collected in the morning after an overnight (8–12 h) fast. Venous blood samples, including serum and plasma were collected from an antecubital vein followed by centrifugation at 3000× *g* for 15 min at 4 °C. Immediately thereafter, serum/plasma samples were frozen and stored at −80 °C prior to analysis. Measurements of plasma glucose, serum insulin and creatinine levels, lipid profiles and fibrinogen levels were performed in a central laboratory as previously reported [36]. The HOMA-IR index was calculated using the formula: HOMA-IR = fasting serum insulin (μU/mL) × fasting plasma glucose (mmol/L)/22.5. Quantitative insulin sensitivity check index (QUICKI), used as the measurement for insulin sensitivity, is defined as follows: QUICKI = 1/[(log(I0) + log(G0)], where I0 is the fasting plasma insulin level (μU/mL) and G0 is the fasting blood glucose level (mg/dL) [37]. The estimated glomerular filtration rate (eGFR) values were determined with the following equation: 194 × serum creatinine^−1.094^ × age^−0.287^ (×0.739 if female) [38].

### 4.4. Assays

Most markers, including serum C-reactive protein (CRP), serum amyloid A (SAA), soluble intercellular adhesive molecule (sICAM1), soluble vascular cell adhesive molecule (sVCAM1), soluble E-selectin (sE-selectin), adiponectin, matrix metalloproteinase 9 (MMP9) and plasma Monocyte chemotactic protein 1 (MCP1) were measured using a sandwich enzyme-linked immunosorbent assay (ELISA) developed in-house. All in-house kits showed good correlation when compared with commercially available ELISA kits [39,40,41]. Circulating serum resistin, lipocalin 2, matrix metalloproteinase 2 (MMP2) and plasma matrix metalloproteinase 1 (MMP1), soluble P-selectin (sP-selectin), soluble tumor necrosis factor receptor II (sTNFRII) and YKL-40 were measured using commercially available ELISA kits from R&D (Minneapolis, MN, USA). Urinary 8-OHdG was measured using a competitive ELISA described elsewhere [42] and was presented as urinary 8-OHdG-to-urinary creatinine ratio.

### 4.5. Statistical Analysis

The chi-square test was used for testing to compare categorical variables of hypertension, diabetes mellitus, metabolic syndrome and smoking. The clinical characteristics that were continuous variables are expressed as means ± SDs and were tested using a two-sided *t*-test or analysis of variance (ANOVA). Pearson correlation coefficients were calculated to determine the association between YKL-40 level and clinical and biochemical factors. Furthermore, a general linear model was applied to capture the major effect of each polymorphism on clinical phenotype variables, with BMI, age, gender and smoking status as confounding covariates. We used dominant models for numeric association test after recoding our SNPs from categorical variables to continuous variables, such as 0, 1 of a particular allele. High-density lipoprotein-cholesterol (HDL-C), low-density lipoprotein-cholesterol (LDL-C), total cholesterol, Triglyceride, CRP, SAA, sICAM1, sVCAM1, sE-selectin, sP-selectin, MMP1, MMP2, MMP9, YKL-40, MCP1, and sTNFRII values were logarithmically transformed prior to statistical analysis to adhere to a normality assumption. In addition, step-wise linear regression analysis was used to determine independent predictors of YKL-40 levels. All calculations were performed with standard statistical SPSS software (SPSS, Chicago, IL, USA). Measures of haplotype frequencies were estimated using the expectation-maximization algorithm implemented in HelixTree Genetics Analysis software (Golden Helix, Bozeman, MT, USA). In the haplotype analysis, Coefficients and *p* values were estimated based on haplotype trend regression analysis implemented in the HelixTree program (Golden Helix). The selected haplotype was compared to all other unselected haplotypes. An analysis of deviation from Hardy–Weinberg equilibrium, estimation of linkage disequilibrium between polymorphisms, association of haplotypes with YKL-40 level was performed using the Golden Helix SVS Win32 7.3.1 software (Golden Helix). Values of *p* < 0.05 using a two-sided test were considered statistically significant.

## 5. Conclusions

Our data confirmed the significant association of circulating YKL-40 level with atherosclerosis-related quantitative traits and the risk of PAD in Taiwanese, suggesting a higher burden of subclinical cardiovascular disease with elevated YKL-40 levels and a possible pathogenetic role of YKL-40 in PAD. In contrast, a significant association between the *CHI3L1* gene variants and YKL-40 levels, but the absence of an association with either atherosclerosis-related quantitative traits or PAD for the *CHI3L1* gene variants suggests that the influence in baseline YKL-40 levels due only to YKL-40 promoter polymorphisms may not be large enough to alter the risk of atherosclerotic cardiovascular disease.

## Supplementary Materials

Supplementary materials can be found at http://www.mdpi.com/1422-0067/15/12/22421/s1.
